# Neural differentiation, selection and transcriptomic profiling of human neuromesodermal progenitor-like cells *in vitro*

**DOI:** 10.1242/dev.166215

**Published:** 2018-07-12

**Authors:** Laure Verrier, Lindsay Davidson, Marek Gierliński, Alwyn Dady, Kate G. Storey

**Affiliations:** 1 Division of Cell and Developmental Biology, School of Life Sciences, University of Dundee, Dow Street, Dundee DD1 5EH, UK; 2 Human Pluripotent Cell Facility, Division of Cell and Developmental Biology, School of Life Sciences, University of Dundee, Dow Street, Dundee DD1 5EH, UK; 3 Data analysis group, Division of Cell and Developmental Biology, School of Life Sciences, University of Dundee, Dow Street, Dundee DD1 5EH, UK

**Keywords:** Neuromesodermal progenitor-like cells, Human neural development, Human spinal cord, Dual SMAD inhibition, CRISPR-Cas9, Human ES cells, *Nkx1.2* reporter, Human neuromesodermal progenitor transcriptome

## Abstract

Robust protocols for directed differentiation of human pluripotent cells are required to determine whether mechanisms operating in model organisms are relevant to our own development. Recent work in vertebrate embryos has identified neuromesodermal progenitors as a bipotent cell population that contributes to paraxial mesoderm and spinal cord. However, precise protocols for *in vitro* differentiation of human spinal cord progenitors are lacking. Informed by signalling in amniote embryos, we show here that transient dual-SMAD inhibition, together with retinoic acid (dSMADi-RA), provides rapid and reproducible induction of human spinal cord progenitors from neuromesodermal progenitor-like cells. Using CRISPR-Cas9 to engineer human embryonic stem cells with a GFP-reporter for neuromesodermal progenitor-associated gene *Nkx1.2* we facilitate selection of this cell population. RNA-sequencing was then used to identify human and conserved neuromesodermal progenitor transcriptional signatures, to validate this differentiation protocol and to reveal new pathways/processes in human neural differentiation. This optimised protocol, novel reporter line and transcriptomic data are useful resources with which to dissect molecular mechanisms regulating human spinal cord generation and allow the scaling-up of distinct cell populations for global analyses, including proteomic, biochemical and chromatin interrogation.

## Abstract (precis)

**Summary:** The first transcriptome profiling of human neuromesodermal progenitor-like cells, generation of human spinal cord progenitors from these cells and a GFP-reporter for Nkx1.2 in human embryonic stem cells.

## INTRODUCTION

**Fig. 1.**
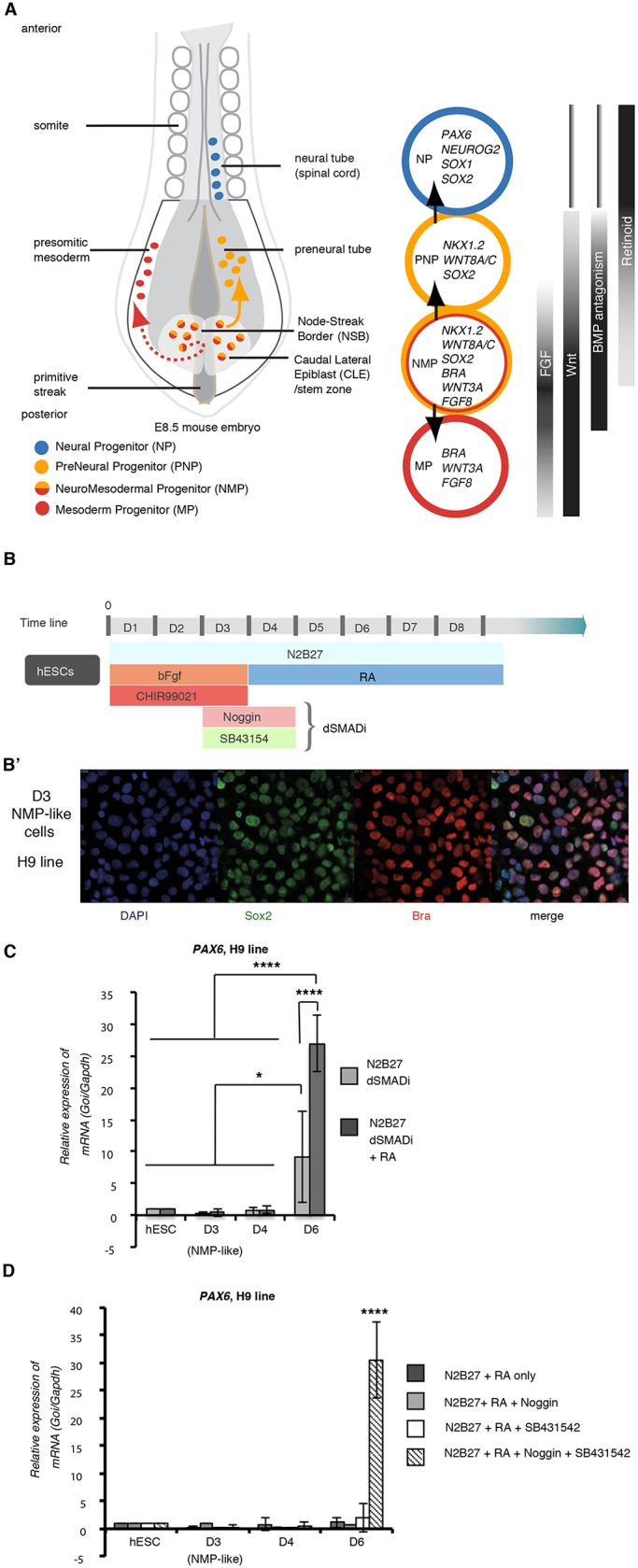
**Protocol for neural differentiation of human NMP-like cells.** (A) Schematic of mouse E8.5 caudal embryo. Selected progenitor cell marker genes and signalling pathways operating during posterior neural differentiation. (B,B′) Schematic of the developed differentiation protocol, including a dual-SMAD inhibition step (dSMADi-RA) (B), and immunocytochemistry for Bra (T) and Sox2 in day 3 NMPs (three independent experiments) (B′). (C) RT-qPCR showing *PAX6* in the H9 cell line differentiated as in B, with or without 100 nM RA from day 3. (D) RT-qPCR for *PAX6* in cells differentiated as in B, with varying SMAD inhibitor inclusion day 2-4. RT-qPCR graphs represent expression normalized to *GAPDH* and relative to hESC levels (three independent experiments, error bars indicate the s.e.m.; ****P<0.001, *P<0.05 (ANOVA test).

Head and trunk nervous systems have distinct developmental origins. Head or anterior neural progenitors are derived from the epiblast rostral to the primitive streak and will form regions of the brain. In contrast, progenitors of trunk or posterior neural tissue (posterior hindbrain and spinal cord) arise from epiblast adjacent to and within the anterior primitive streak [known as caudal lateral epiblast (CLE) and node streak border (NSB), respectively] (Wilson et al., 2009) (Fig. 1A). In recent years, evidence has accrued which indicates that, unlike anterior, posterior neural tissue is generated via an intermediary neuromesodermal progenitor (NMP), which contributes to paraxial mesoderm as well as to posterior neural tube (reviewed by Tzouanacou et al., 2009; Gouti et al., 2015; Henrique et al., 2015; Tsakiridis and Wilson, 2015). Human, mouse and chick embryos, as well as *in vitro* NMPs, are identified by co-expression of early neural (Sox2) and mesodermal brachyury (Bra, T) proteins, but as yet lack unique molecular markers (Olivera-Martinez et al., 2012; Gouti et al., 2014; Turner et al., 2014; Henrique et al., 2015; Tsakiridis and Wilson, 2015). Although we are beginning to uncover how mouse NMPs are regulated, human NMP-like cells and their derivatives are less well characterised, in part because this requires creation of robust *in vitro* models.

Most *in vitro* differentiation protocols are informed by our understanding of how the cell type of interest is generated during embryonic development. In the caudal end of amniote embryos, FGF and Wnt signalling act in a positive-feedback loop to maintain the elongation of the body axis (Aulehla et al., 2003; Olivera-Martinez and Storey, 2007; Wilson et al., 2009). FGF signalling also promotes expression of genes characteristic of CLE, including the transcription factor *Nkx1.2* (Delfino-Machin et al., 2005; Sasai et al., 2014). *Nkx1.2* expression extends into the preneural tube (PNT) (Spann et al., 1994; Schubert et al., 1995; Rodrigo-Albors et al., 2016 preprint). Here, preneural progenitors (PNPs) downregulate *Bra* (*T*) transcribe the early neural gene *Sox2*, but as yet do not express neurogenic genes such as *Neurog2* and *Pax6* (Scardigli et al., 2001; Scardigli et al., 2003; Bel-Vialar et al., 2007) (Fig. 1A). Retinoic acid synthesized in neighbouring paraxial mesoderm mediates the transition from PNPs, repressing expression of *Fgf8*, *Wnt8a*, *Wnt8c* and *Wnt3a* (Shum et al., 1999; Diez del Corral et al., 2003; Sirbu and Duester, 2006; Olivera-Martinez and Storey, 2007; Cunningham et al., 2015), and is then further required for neurogenic gene transcription (Diez del Corral et al., 2003; Ribes et al., 2008).

In addition to the involvement of these signalling pathways in NMP regulation, inhibition of BMP signalling is required for *Sox2* transcription in the CLE/NSB (Takemoto et al., 2006). In mouse and chick embryos, various BMP and TGFβ antagonists (noggin, chordin and follistatin) are expressed in the anterior primitive streak, emerging notochord and newly formed somites close to posterior neural tissue (Albano et al., 1994; Liem et al., 2000; Chapman et al., 2002). When considered together with the requirement for BMP antagonism in anterior neural induction (Hemmati-Brivanlou and Melton, 1997; Harland, 2000; Kuroda et al., 2004; Linker and Stern, 2004), the experiments of Takemoto et al. indicate an ongoing requirement for BMP antagonism during the progressive generation of the posterior nervous system.

Almost all *in vitro* protocols for making NMP or NMP-like cells from mouse and human embryonic stem cells (hESCs) involve exposure to a Wnt agonist over different time periods with or without FGF (Gouti et al., 2014; Tsakiridis et al., 2014; Turner et al., 2014; Lippmann et al., 2015); one approach has included TGFβ inhibition (to promote loss of self-renewal in human ESCs and repress mesendoderm differentiation; Chambers et al., 2009; Denham et al., 2015). It is well established that efficient induction of anterior neural tissue from hESCs is achieved by exposure to inhibitors of both TGFβ and BMP signalling (known as dual-SMAD inhibition) (Chambers et al., 2009). However, a role for BMP inhibition in the differentiation of neural tissue from NMPs *in vitro* has not been assessed. Here, we show that neural differentiation from human NMP-like cells is promoted by transient dual-SMAD inhibition. We deploy CRISPR-Cas9 engineering to make a reporter for enrichment for human NMP-like cells and provide the first transcriptomic profiling of this cell population and the derived spinal cord progenitors.

## RESULTS AND DISCUSSION

### Robust differentiation of human NMP-like cells into posterior neural progenitors by inclusion of transient dual SMAD inhibition

In human ESCs, the simplest approach to make NMP-like cells involves removal of self-renewal conditions and exposure to FGF and the Wnt agonist CHIR99021 for 3 days. The cells generated in this way were then differentiated into neural progenitors by day 6, following replating and culture in basal media alone (Gouti et al., 2014). We first assessed the reproducibility of this protocol to generate *PAX6*-expressing neural progenitors. Culturing hESCs in neurobasal medium supplemented with 1× N2, 1× B27 medium bFGF (20 ng ml^−1^) and CHIR99021 (3 µM) for 3 days readily generated Sox2/Bra (T) co-expressing NMP-like cells (Fig. S1A,B). However, subsequent differentiation after cell dissociation and re-plating in just neurobasal medium/1× N2/1× B27 at the end of day (D) 3, did not generate *PAX6*-positive cells by end of day 6 (D6) (assessed in two hESC lines, SA121 and H9) (Fig. S1C,D). We then carried out a series of experiments aimed at inducing prompt neural differentiation, as indicated by expression of *PAX6* by D6. Introduction of all-trans retinoic acid (RA) 100 nM from the beginning of the neural differentiation protocol on D4 was not sufficient in either cell line (Fig. S1C,D). This inability to induce prompt *PAX6* expression from NMP-like cells might reflect inherent differences between hESC lines, but may also involve variant culture conditions, including the extent of cell dissociation on re-plating following NMP-like cell induction. This may influence cell-cell signalling and could mimic inhibition of BMP signalling, as reported on dissociation of *Xenopus* animal cap ectoderm (Wilson and Hemmati-Brivanlou, 1995). Furthermore, exposure to dual SMAD inhibitors (dSMADi) and therefore attenuation of BMP and TGFβ receptor type 1 signalling, is known to promote anterior neural differentiation of hESCs following removal of self-renewal conditions (Chambers et al., 2009). Informed by the timing of exposure to endogenous TGFβ inhibitors experienced by cells in the CLE and PNT in the amniote embryo (Fig. 1A), we next introduced Noggin 50 ng ml^−1^ and the TGFβ receptor type 1 inhibitor SB431542 (10 µM) from the beginning of D3 to the end of D4. This step did not alter induction of NMP-like cells on D3 (Fig. 1B′ and see flow cytometry data Fig. S2) and in combination with subsequent exposure to RA from D4, robust *PAX6* expression was induced by D6 (Fig. 1C). Importantly, inclusion of either Noggin or SB431542 alone with RA was not effective (Fig. 1D), indicating that dual SMAD inhibition is required to augment neural differentiation in this context. The reproducibility of this protocol (Fig. 1B) was further demonstrated by rapid induction of *PAX6* in a hiPSC line (Fig. S3, ChiPS4).

**Fig. 2.**
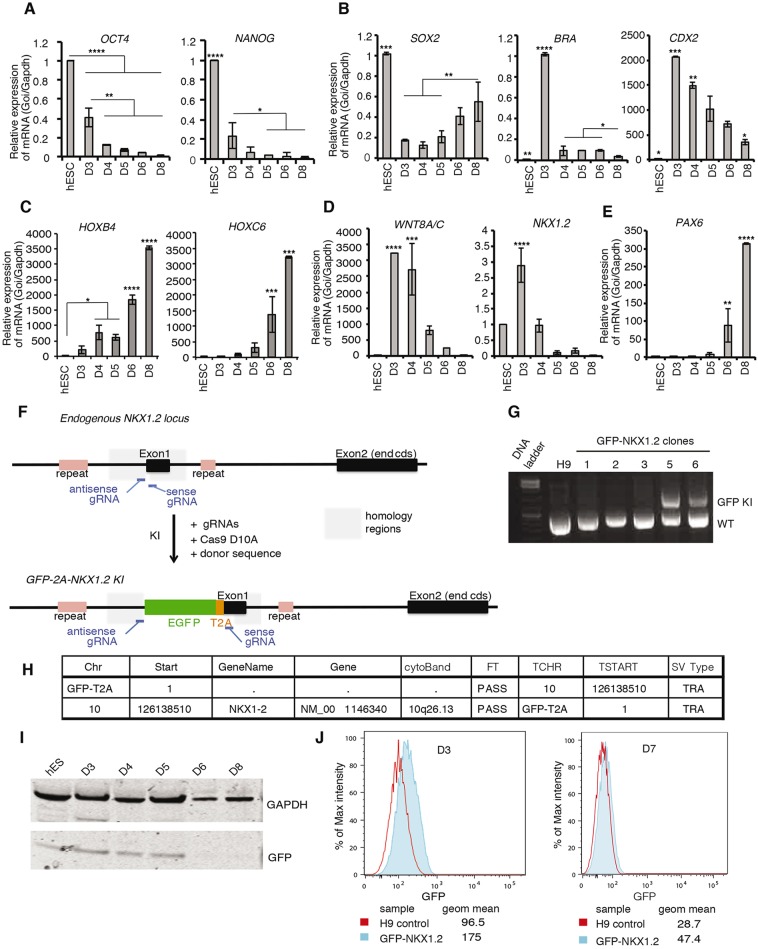
**RT-qPCR for selected genes during dSMADi-RA differentiation and generation of a GFP-Nkx1.2 reporter line.** (A-E) RT-qPCR assessing relative expression of key marker genes in H9 cells exposed to the dSMADi-RA protocol (Fig. 1F). (A) Declining expression of the pluripotency genes *OCT4* and *NANOG*. (B) *SOX2*, *BRA* (*T*) and *CDX2* expression dynamics. (C) *HOXB4* and *HOXC6* during differentiation. (D) Expression of the neural progenitor marker *PAX6*. (E) *WNT8A*/*C* and *NKX1*.*2*, which are characteristic of preneural progenitors and NMPs. ****P<0.0001, ***P<0.001, **P<0.01, *P<0.05 (ANOVA test). (F) Experimental strategy schematic: H9 hESCs were engineered using CRISPR/Cas9, knocking-in the GFP-T2A sequence upstream of exon 1 in *NKX1.2*. Positions of the gRNAs and homologous regions used in the repair template are indicated. (G) PCR amplification of the *NKX1.2* locus using primers framing the insertion site. H9, untransfected control; 1-3, GFP-negative clones; 5 and 6, clones containing the GFP insertion (GFP KI, knock-in; WT, wild-type allele). (H) Whole-genome sequencing of GFP-Nkx1.2 clone 5. Structural variation analysis relative to GFP-T2A sequence: FT, per sample genotype filter; TCHR, chromosome for the translocation breakpoint coordinate; TSTART, translocation breakpoint coordinate; SV type, structural variation type; TRA, translocation. (I) Western blot of GFP during differentiation of the GFP-Nkx1.2 line. (J) Flow cytometry of GFP expression at day 3 and day 7. The percentage of maximum intensity for the GFP-channel is plotted. Data are representative of at least two experiments.

To characterize this dSMADi-RA differentiation protocol, we analysed the expression dynamics of key cell state marker genes using quantitative reverse transcription PCR (RT-qPCR). Pluripotency genes *NANOG* and *OCT4* were dramatically reduced from hESC to D3(NMP-like) and transcripts were lost quickly as these cells differentiated (Fig. 2A), as observed in mouse and chick embryo and mouse ESC-derived NMPs (Tsakiridis et al., 2014; Gouti et al., 2014). D3 (NMPs) were characterized by high levels of *BRA* (*T*) and *CDX2* transcription (Fig. 2B). As in mouse ESC-derived NMPs, *SOX2* transcripts were lower in D3 (NMP-like cells) than in hESCs, despite high levels of Sox2 protein in NMPs (Gouti et al., 2014; Turner et al., 2014) (Figs 1F and 2B). Cdx genes regulate signalling that maintains the mouse NMP cell state and also induce expression of posterior Hox genes, which confer anterior-posterior identity (Young and Deschamps, 2009; Young et al., 2009; Gouti et al., 2017). NMP-like cells expressed *HOXB4* and little *HOXC6* (Fig. 2C) and, together with subsequent RNASeq analysis (see below) that revealed transcription of Hox gene paralogues only within a range from a1 to a7 on D3, this suggests that these cells are equivalent to mouse embryo E7.5-8.5 CLE/NSB cells, which co-express Hox genes across this range (Huang et al., 1998; Yu et al., 1998). The anterior boundary of *Hoxa7* defines the cervical/thoracic boundary at later stages, suggesting that human NMP-like cells and their derivatives generated with this protocol possess an anteroposterior identity in this region (Fig. 2C). In the embryo, differentiation from NMPs to neural progenitors involves downregulation of *Bra* (*T*) and entry into a transitional preneural cell state (Fig. 1A), which is characterized by persisting expression of *WNT8A/C* and *NKX1.2* (Fig. 2D). As the expression of these genes declines, *PAX6* is then transcribed, rising to a peak at D8 (Fig. 2E). This suggests that neural progenitors arise between D5 and D8. This protocol therefore provides an assay with which to investigate the human NMP-like cell state and how this alters to form spinal cord progenitors.

### Generation of a human Nkx1.2 reporter cell line

Cell populations generated *in vitro* are inevitably heterogeneous, so we next made a reporter line that could be used to enrich for NMP-like cells. We took advantage of CRISPR/Cas9 technology (Komor et al., 2017) to engineer H9 hESCs to express GFP under the control of the endogenous *NKX1.2* promoter. This homeodomain-containing transcription factor is highly expressed in NMPs (CLE and NSB) in the mouse embryo and is detected at lower levels in the cells becoming neural progenitors (preneural cells) or in cells ingressing into the primitive streak; it is then lost in neural and mesodermal progenitors (Figs 1A and 2D) (Spann et al., 1994; Schubert et al., 1995; Rodrigo-Albors et al., 2016 preprint). We reasoned that selection for high *NKX1.2* expression at D3, when *Bra* (*T*) transcripts are high, would enrich for NMP-like cells. Towards this aim, a GFP-T2A sequence (Kim et al., 2011) was knocked-in to the *NKX1.2* locus in-frame just upstream of exon 1 (Fig. 2F and see Materials and Methods). Correct targeting was confirmed by PCR across the integration site and subsequent fragment sequencing (Fig. 2G, Fig. S4). Whole-genome sequencing and structural variation analysis of these data further confirmed that the *NKX1.2* gene was the only locus modified by integration of GFP-T2A (Fig. 2H). Using the CRISPR-Cas9 approach, we thus generated a GFP-NKX1.2 hESC line bearing a mono-allelic insertion of the GFP-T2A specifically in the *NKX1.2* locus.

Differentiation of this GFP-NKX1.2 reporter line using the dSMADi-RA protocol was then characterized by western blot; revealing GFP expression up to day 5 (Fig. 2I), including low-level GFP in hESC (consistent with detection of *NKX1.2* in H9 hESCs) (Fig. 2E). Flow cytometry (without GFP antibody) further confirmed GFP expression at D3 in GFP-NKX1.2 cells compared with the auto-fluorescence profile of wild-type H9 cells differentiated in parallel, which was then lost as cells differentiate (at D7) (Fig. 2J). To confirm that *Nkx1.2* locus modification did not impair differentiation, we used immunocytochemistry and flow cytometry to assess SOX2/BRA (T) co-expression on D3 (Fig. S2) and RT-qPCR (Fig. S5) to profile expression of marker genes during dSMADi-RA differentiation. These analyses indicated that the engineered line made NMP-like cells and that its differentiation was comparable with that of the parental H9 line (Figs S2 and S5, Fig. 2A-E). Similar results were obtained with a second GFP-NKX1.2 line, demonstrating the reproducibility of this approach (Fig. S6).

### Identity and conservation of human NMP transcriptional signature

**Fig. 3.**
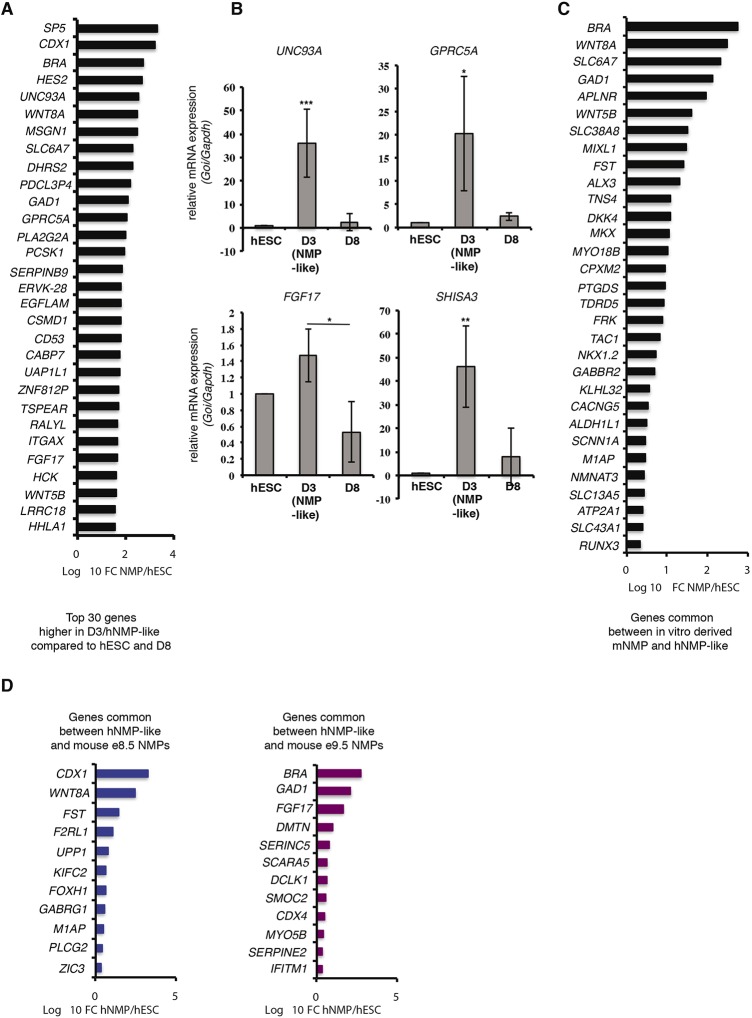
**Characterization and conservation of the human D3(NMP-like cell) transcriptome.** (A) Genes preferentially expressed in human D3(NMP-like cells) compared with hESC and hD8 neural progenitors (NPs). Genes were considered to be preferentially expressed in hD3 NMP-like cells when there was a greater than twofold change between hD3 and both hESCs and hD8 NPs (Table S1). (B) RT-qPCR for subset of D3-enriched genes. ***P<0.001, **P<0.01, *P<0.05 (ANOVA test). (C) Comparison of human NMP-like-enriched genes (this study) and bulk-RNA-seq of mESC-derived NMPs (Gouti et al., 2014). (D) Comparison of NMP-like-enriched genes in human (this study) with mouse embryo eNMP transcriptional signatures obtained by comparing scRNA-seq data for E8.5 and E9.5 mouse embryos (Gouti et al., 2017).

We next used this GFP-NKX1.2 cell line to select for high GFP-expressing cells on D3 using FACS (see Materials and Methods) and generated RNA-seq data for D3. This was compared with RNA-seq data for D8 NPs (not subjected to prior selection) and published RNA-seq data for H9 hESCs (Chu et al., 2016). This included not only expected NMP-associated genes *BRA* (*T*), *CDX1, SP5, WNT8A/C* and *FGF17*, but also new genes, such as *UNC93*, which encodes a membrane protein of unknown function, and *GPRC5A*, a gene encoding an orphan G-protein-coupled receptor responsive to retinoid signalling (Cheng and Lotan, 1998). Some enriched genes (*FGF17*, *GPRC5A* and *UNC93A*) were then validated by RT-qPCR, including a gene not in the top list (*SHISHA3*), which attenuates FGF and Wnt signalling (Yamamoto et al., 2005) (Fig. 3B).

This human D3-NMP-like gene list was next compared with that for genes uniquely upregulated in *in-vitro*-derived mouse NMPs (Gouti et al., 2014). This identified 31 conserved genes (Fig. 3C). These include not only transcription factors known to be expressed in mouse NMPs, e.g. *BRA *(*T*)*, NKX1.2* and *MIXL1*, but also newly implicates *MKX* (mohawk/*IRX1L*) (Liu et al., 2006), *ALX3* (Beverdam and Meijlink, 2001) and *RUNX3* as transcriptional regulators. Predicted signalling pathways, Wnt (*WNT8A*, *WNT5A*, *DKK4*) and TGFβ antagonism (*FST*, follistatin) were also represented, along with genes involved in new signalling activities. These include four solute carriers (*SLC13A5*, *SLC38A8*, *SLC43A1* and *SLC6A7*). *SLC6A7* is a member of the gamma-aminobutyric acid (GABA) neurotransmitter gene family and two further genes mediating GABA signalling are also conserved: *GAD1* (glutamic acid decarboxylase), which synthesizes GABA from glutamate and is transcribed in the mouse tailbud (Maddox and Condie, 2001); and GABA receptor *GABBR2*/*GPRC3B*. In neurons, GABA-B receptors can trigger inactivation of voltage-gated calcium channels (Padgett and Slesinger, 2010). Two further conserved NMP genes, *CACNA1C* [a calcium-channel auxiliary subunit/CaV1.2 implicated in maintaining calcium-channel inactivation (Soldatov et al., 1997)] and *ATP2A1* [a calcium transporting ATPase that maintains low cytoplasmic calcium (Shull et al., 2003)], may additionally operate via different mechanisms to restrict intracellular calcium. This is consistent with the requirement for calcium signalling in neural induction, as indicated by *SOX2* transcription in chick embryos (Papanayotou et al., 2013). Indeed, *Sox2* transcripts are characteristically low in mNMPs (Gouti et al., 2014). To test this predicted increase in calcium signalling during neural differentiation, we assessed this in D3(NMP-like) cells and D8 neural progenitors using a fluorescence-based reporter (Fluo3-AM) that binds free intracellular Ca^2+^ (Tsien, 1981). This revealed elevated calcium signalling in neural progenitors cells in comparison with NMP-like cells (Fig. S7).

As there are not only species differences between these data sets, but also *in vitro* protocol variation, we additionally compared the human D3/NMP-like molecular signature with those obtained for mouse embryonic NMPs at E8.5 and E9.5 using single-cell RNA-seq (Gouti et al., 2017). This identified 23 conserved genes (Fig. 3D) and, again, included *GAD1* and another GABA receptor, *GABRG1*, which belongs to the type-A family, shown to regulate stem cell proliferation (Andäng et al., 2008). GABA biosynthesis is an output of the tricarboxylic acid (TCA) cycle, input to which can come from glycolytic metabolism, which was recently shown to operate in tailbud progenitor cells (Bulusu et al., 2017; Oginuma et al., 2017). It will therefore be important in the future to understand the relationship between this metabolic state and GABA production in axial progenitors (Fig. 3D).

### Transcriptomic characterization of the differentiation protocol

**Fig. 4.**
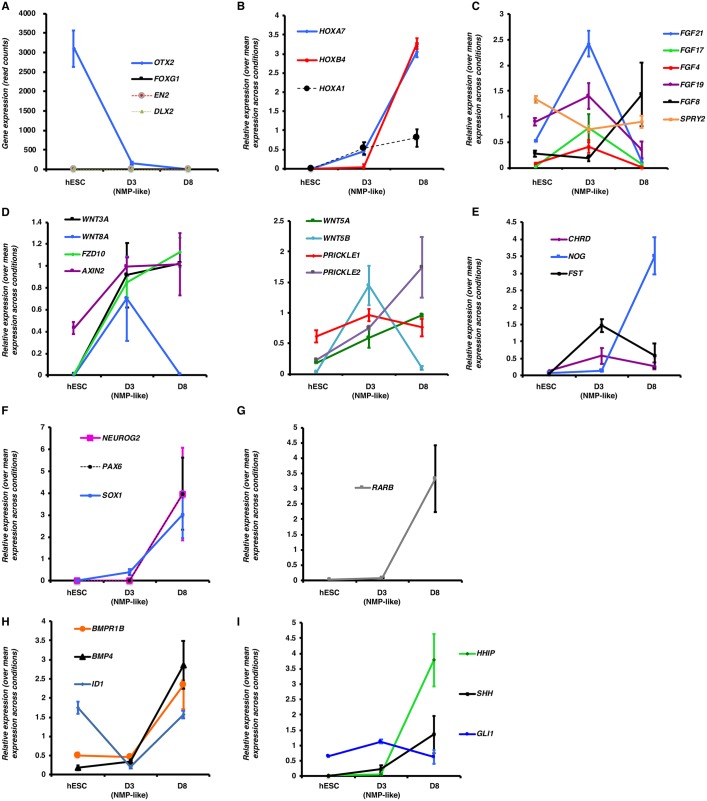
**Expression of selected genes across three conditions analysed by RNA-seq.** (A) Anterior neural marker genes, presented as read counts. (B) Main Hox genes expressed at D3 and D8. (C,D) Selected components of (C) FGF and (D) Wnt signalling pathways. (E) Selected BMP/TGFβ inhibitors. (F) Neural progenitor and neurogenic genes. (G) Retinoid receptor β during human NMP-like cell differentiation. (H,I) Selected components of (H) BMP and (I) Shh signalling pathways. (B-I) Relative expression of each gene is normalized to its mean expression across all conditions±s.e.m. for each gene are shown.

These RNA-seq data also helped to characterize cell types generated with the dSMADi-RA differentiation protocol. The mesendoderm marker *SOX17* was not detected, nor were transcripts from anterior neural genes (*FOXG1*, *EN2 *and* DLX2*) in any condition (<10 reads), whereas *OTX2*, which is initially expressed in the early epiblast and primitive streak (Ang et al., 1996; Henrique et al., 2015), declines sharply from hESCs (Fig. 4A). This is not surprising given hESC exposure to FGF and Wnt signalling for 3 days to generate NMP-like cells, at which time cells begin to express a range of Hox genes, including *HOXA1*, *HOXB4 and HOXA7* (Fig. 4B). In this assay, therefore, NMP-like cells possess a posterior identity prior to their progress along the neural differentiation pathway. Components of signalling pathways known to regulate embryonic NMPs (reviewed by Henrique et al., 2015) exhibited expected gene expression profiles (Fig. 4C-E). High-level transcription of neural progenitor and neurogenic genes (Fig. 4F) was detected on D8 and correlated with increased retinoid signalling reported by *RARB* transcription (Fig. 4G). The expression of both BMP and Shh pathway genes (Fig. 4H,I) on D8 suggested that induced spinal cord progenitors are exposed to dorsal (BMP) and ventral (Shh) patterning signals. However, although dorsal neural progenitor and neural crest associated genes were expressed along with some more-ventral progenitor genes (Table 1), the ventral-most marker *NKX2.2* and the floor plate marker *FOXA2* were not detected at D8. The early transcription of neural crest genes in this differentiation assay further suggests that, as in the elongated embryonic body axis and in mouse ES-derived *in vitro* spinal cord assays, dorsal progenitor cell types emerge prior to ventral progenitors (Meinhardt et al., 2014).

**Table 1.**
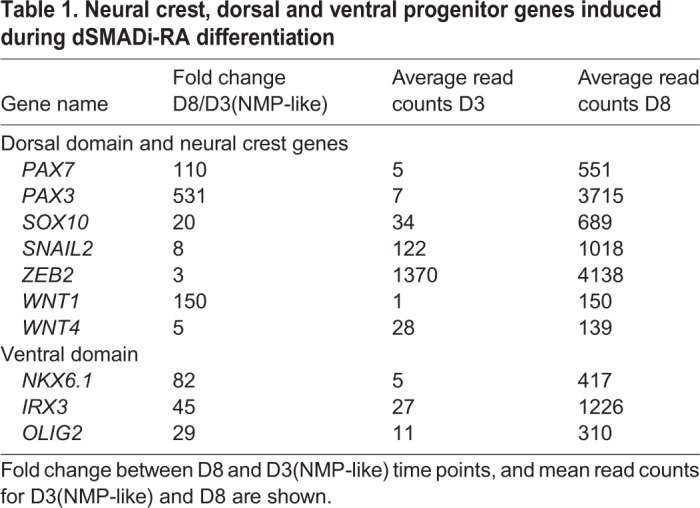
**Neural crest, dorsal and ventral progenitor genes induced during dSMADi-RA differentiation**

To establish whether ventral cell types, such as motoneurons, can be derived from D3(NMP-like) cells, we further adapted the neural differentiation regime (Amoroso et al., 2013), including extension of the culture period to 21 days. This reproducibly generated motorneurons, identified as cells co-expressing islet 1 and HB9 (Fig. S8).

Guided by signalling in model vertebrate embryos, we have devised a protocol for the robust differentiation of human spinal cord progenitors from NMP-like cells, which could be further differentiated into expected spinal cord cell types, such as motorneurons. This protocol can be used for future mechanistic and translational approaches, including development of human neuroepithelial cell behaviour assays. The GFP-NKX1.2 reporter line allowed selection of cells expressing high levels of *NKX1.2* on D3 and has the potential to be further engineered to report for *BRA *(*T*)**, select for later NKX1.2^+^/BRA (T)^−^ cells and thus identify early changes in neural differentiation. These RNA-seq data not only served to validate this differentiation protocol and uncover a conserved NMP-like transcriptional signature, but also identified potential new signalling pathways, including those mediated by GABA and calcium, involved in the regulation of the NMP cell state.

## MATERIALS AND METHODS

### Human ES cell culture and differentiation

Human ES cells (H9, WiCell; SA81 and SA121, Cellartis AB) and human iPS cells (ChiPS4, Cellartis AB) were maintained as feeder-free cultures in DEF medium (Cellartis AB) supplemented with bFGF (30 ng/ml, Peprotech) and Noggin (10 ng/ml, Peprotech) on fibronectin- (Millipore, 5 µg cm^−2^) coated plates, and enzymatically passaged to single cells using TrypLE select (Life Technologies) according to the manufacturer's recommendations. Metadata for quality control and passage numbers for all pluripotent stem cells (PSC) used in this study are provided in the supplementary Materials and Methods. For single-cell passaging, the medium was supplemented by addition of the Rho kinase inhibitor Y27632 (10 µM, Tocris). All experiments with hESCs were approved by the UK Stem Cell Bank steering committee (licence numbers SCSC14-28 and SCSC14-29).

For differentiation assays, PSCs were plated on Geltrex matrix (20 µg cm^−2^, Life Technologies) at a density of 4×10^4^ cells cm^−2^ in DEF medium supplemented with bFGF, Noggin and Y-27632 as above, and cells were allowed to attach for 24 h. To start differentiation, the medium was changed to neurobasal medium supplemented with 1× N2 and 1× B27 supplements (all Life Technologies), and Chiron99021 (3 µM, Tocris) and bFGF (20 ng ml^−1^), and cells were incubated for 48 h. The medium was then changed to neurobasal medium supplemented with 1× N2, 1× B27, Chiron99021 (3 µM, Tocris), bFGF (20 ng ml^−1^), Noggin (50 ng ml^−1^) and SB431542 (10 µM, Tocris), and cells were incubated for a further 24 h to obtain NMP-like cells.

For further differentiation, NMP-like cells were dispersed using PBS-EDTA 0.5 mM and seeded at a density of 2×10^5^ cells cm^−2^ on Geltrex matrix (20 µg cm^−2^) in neurobasal medium supplemented with 1× B27, 1× N2, all-trans retinoic acid (100 nM, Sigma-Aldrich) and Y-27632 (10 µM, Tocris), and allowed to attach overnight. Cells were then cultured in neurobasal medium supplemented with 1× N2, 1× B27 and all-trans retinoic acid (100 nM) for the indicated time to obtain later stage progenitors.

NMP-like cells were differentiated into motorneurons using a protocol adapted from Amoroso et al. (2013). Briefly, D3(NMP-like) cells were replated as described above and allowed to attach overnight. The medium was then changed to neurobasal medium supplemented with 1× N2, 1× B27, all-trans retinoic acid (100 nM), L-ascorbic acid 2-phosphate (400 nM, Sigma-Aldrich) and BDNF (20 ng ml^−1^, Peprotech), and the cells cultured for 48 h. The medium was then further supplemented by the addition of C25Il Shh (20 ng ml^−1^, Dundee Cell Products) and cells cultured for 17 days changing the medium every 48 h.

### RT-qPCR

Total RNA was extracted using the RNEasy mini kit (Qiagen), following the manufacturer's instructions, with the addition of a DNAse digestion step performed on the column for 15 min with RQ1-DNase (Promega). After initial denaturation for 5 min at 70°C in presence of 1 µg random primers, 500 ng of RNA per sample were reverse transcribed for 1 h in 20 µl reaction volume containing 0.5 mM dNTPs, 5 mM MgCl2, 1× ImProm-II RT buffer, 20 U RNasin and 160 U of ImProm-II RT (Promega). Samples were incubated for 15 min at 70°C to stop the reaction. qPCR analysis was performed using primers described in Table S2 on either a Mastercycler RealPlex2 (Eppendorf) or an AriaMX (Agilent) device in presence of PerfeCTa SYBR Green SuperMix for iQ (Quanta Biosciences) or BrilliantIII SYBRgreen PCR MasterMix (Agilent), respectively. Relative expression was calculated using the ΔΔCt method, normalizing each gene of interest to *Gapdh* levels.

### Western blot

Western blots were performed using standard protocols. Briefly, proteins were extracted using RIPA buffer [150 mM sodium chloride, 1.0% Triton X-100, 0.5% sodium deoxycholate, 0.1% SDS (sodium dodecyl sulphate) and 50 mM Tris (pH 8.0)]. Cell extract was incubated on ice for 30 min in presence of DNAse (Universal Nuclease, Pierce) and spun down for 20 min at full speed. Protein concentration in supernatant was determined using a Bradford Assay with a BSA standard curve ranging from 0-2 mg/ml. The samples were diluted in NuPage 4× sample buffer (Life Technologies) and loaded onto a 4-12% gradient gel (Novex NuPAGE, Life Technologies). Western blots were performed using standard procedures and antibodies used at the following concentrations: anti-GAPDH 1 µg/ml (ab9484, Abcam) and anti-GFP 1 µg/ml (ab6673, Abcam). Detection was performed with anti-goat Dylight 6800 conjugate (1:10,000, Life Technologies) and anti-mouse DyLight 800 conjugate (1:10,000, Life Technologies) on a LI-COR imaging device (BioSciences).

### Immunofluorescence microscopy

Cells were fixed by adding formaldehyde to a final concentration of 3.7% in PBS, then permeabilized and blocked in PBS/0.1% TritonX-100/4% (w/v) BSA. Incubation was performed at 4°C overnight with primary antibodies at the following concentrations: goat anti-brachyury 1 µg ml^−1^ (AF2085, R&D), rabbit anti-Sox2 5 µg m^−1^ (ab5603, Millipore), rabbit anti-β-III-tubulin 1 µg ml^−1^ (T2200, Sigma-Aldrich), mouse anti-HB9 1.75 µg ml^−1^ (81.5C10, Developmental Studies Hybridoma Bank) and rabbit anti-islet 1 2.5 µg ml^−1^ (ab20670, Abcam). Fluorochrome-conjugated secondary antibodies used were the following: anti-goat Alexa647-conjugated 4 µg ml^−1^ (A21447, Invitrogen), anti-rabbit Alexa488-conjugated 4 µg ml^−1^ (A21206, Molecular Probes) and anti-mouse Alexa594-conjugated 4 µg ml^−1^ (A11032, Molecular Probes). Observations were carried out with a DeltaVision fluorescence microscope (GE Healthcare) and images were acquired using softWoRx software, except images in Fig. S8, which were captured on a Zeiss LSM 710 confocal microscope.

### Flow cytometry analysis of protein expression profile

Cells were harvested using TryLEselect, fixed for 10 min in 4% paraformaldehyde and re-suspended as single cells in PBS containing 1% BSA. An additional 10 min methanol fixation step was added for Sox2 and brachyury detection. Primary antibodies were incubated for 1 h at room temperature in PBS containing 4% BSA; cells were then washed and incubation with secondary antibodies carried out for 30 min at room temperature. Antibody used were as follows: goat anti-brachyury 1 µg/ml (AF2085, R&D), rabbit anti-Sox2 5 µg/ml (ab5603, Millipore), anti-goat Alexa647-conjugated 2 µg/ml (A21447, Invitrogen) and anti-rabbit Alexa488-conjugated 2 µg/ml (A21206, Molecular Probes). After washes, fluorescence was measured on a FACSCanto cytometer (BD Biosciences) and results analysed using FlowJo software. Quadrant gates used to estimate the percentage of positive cells were designed based on fluorescence levels detected in the control samples processed without primary antibodies.

### GFP-Nkx1.2 engineering

The donor plasmid construct pDonorNkx1.2NterKI was synthesized by GeneArt. The vector is based on a pMK-RQ backbone and contains a kanamycin-resistance cassette and the GFP-T2A insert flanked by 500 bp homology arms for recombination to the NKX1.2 5′ end. The second plasmid used, px335Nkx1.2NterKIas, encoded the Cas9D10A nickase (Cong et al., 2013) and the antisense gRNA (asgRNA GCCCACGGGCCGGCGGTCGG). A third plasmid, pBABEDpU6Nkx1.2NterKIs, included the sense gRNA (sgRNA GCTGGCATGGCAGGACGGCG) and a puromycin-resistance cassette to select transfected cells. CRISPR-Cas9 mediated gene targeting was performed as follows: H9 hESC were dispersed to single cells using TryLE select (Life Technologies) and re-suspended in DEF medium in the presence of Y-27632 (10 mM, Tocris). For transfection, 5×10^6^ cells were pelleted by centrifugation at 300 ***g*** for 3 min, washed with PBS and re-suspended in 100 µl buffer R (Neon Transfection Kit, Life Technologies) containing 4µg of pDonorNkx1.2NterKI, 2 µg of px335Nkx1.2NterKIas and 2 µg of pBABEDpU6Nkx1.2NterKIs. Electroporation was performed with the Neon Transfection System (Life Technologies) using the following parameters: one pulse, 1150V, 30 ms. Transfected cells were plated and allowed to recover for 36 h, puromycin selection (1 µg ml^−1^) was applied for a further 36 h. Clones were left to grow until easily visible, hand-picked and seeded back in 96-well plates before being amplified. Screening of the clones for GFP integration was performed by PCR using primers amplifying across the insertion sites (GFPcheckFw1+GFPcheckRev1) (Fig. 2G). Correctly targeted integration of the GFP-T2A sequence was checked in 40 transformed hESC clones by PCR across the integration site. Overlapping PCR amplification products spanning the locus from outside the homologous region to inside the GFP sequence on both sides of the integration were sequenced (GFPcheckFw1+GFPcheckRev2 and GFPcheckFw2+GFPcheckRev1). Five clones were found to include the GFP-T2A sequence at the correct locus and these were all heterozygous for GFP-NKX1.2 (Fig. 2G). PCR bands obtained for GFP-NKX1.2 clone 5 were sequenced to check for integrity of the recombination borders and absence of mutations. Results were combined and detailed sequence of the engineered allele was obtained (Fig. S4). Primers used were as follows: GFPcheckFw1, CAGTTGCATCCCCAAGTCTAAGG; GFPcheckFw2, AGTGGAAGCAAAAGACTGAGAGTC; GFPcheckRev1, TTTCTGTGGGTCCAGGATGTCCA; and GFPcheckRev2, GTTGAGTCTGGGGAGCTTGAGC.

### Whole-genome sequencing

gDNA was extracted from GFP-Nkx1.2 hES cells using the DNeasy Blood and Tissue Kit (Qiagen), according to the manufacturer's instruction. Whole-genome sequencing was performed by Novogene and deposited in ENA under accession number PRJEB27242). Briefly, a library was generated from 1 µg gDNA using Truseq Nano DNA HT sample preparation Kit (Illumina) following manufacturer's recommendations and sequenced on an Illumina platform. After quality control, BWA (version 0.7.8-r455) was used to align reads to the genome, using the 1000Genomes (GRCh37+decoy) human as reference. BAM files were sorted using SAMtools (version 1.0) and read duplicates identified using Picard (version 1.111). Structural variation (SV) analysis was carried out using Delly (version 0.7.2) (Rausch et al., 2012), and ANNOVAR (version 2015Mar22) was used to annotate the SV. An average coverage of 33× was obtained (depth exceeded 20× for 92% of bases).

### Cell purification for RNA-seq analysis by FACS

Cells were sorted on a BD Influx (Becton Dickinson) cell sorter using the 100 µm nozzle. FSC versus SSC was used to identify live cells and then FSC-A versus FSC-W to identify single cells. The GFP-positive cells were identified using 488 nm laser light and the parameters GFP (530/40) and PE (580/30). The gate to identify GFP-positive cells was set using a GFP-negative control (H9 cells differentiated in parallel) and events that fell into this gate were sorted to more than 97% purity. 1.5 million GFP-positive cells sorted at day 3 were used per sample for RNA extraction.

### Library preparation for RNA-seq and sequencing

Total RNA was extracted using the RNEasy mini kit (Qiagen), following the manufacturer's instructions, with the addition of a DNAse digestion step performed on the column for 15 min with RQ1-DNase (Promega). RNA concentration was measured on a Qubit device using Qubit RNA BR assay kit (ThermoFisher) and quality was checked on a TapeStation instrument (Agilent). Individually labelled libraries were prepared from 1 µg of RNA per sample using the TruSeq Stranded mRNA Library prep kit (Illumina), according to manufacturer's instructions. Spike-ins were added: 2 µl of a 1/100 dilution ERCC Spike-in controls Mix1 per sample. Libraries were pooled and sequencing was performed on a NextSeq (Illumina) at the Tayside Centre for Genomic Analysis (Ninewells, Dundee, UK) as follows: high output run, 2×75 bp paired end sequencing, between 35 and 46 million uniquely mapped reads obtained per sample (12 samples multiplexed). RNA-seq data are available in the ArrayExpress database (www.ebi.ac.uk/arrayexpress) under accession number E-MTAB-6680.

### RNA-seq analysis

RNA-seq reads were mapped to the reference genome (version GRCh38, release 87) using STAR 2.5.2b, using stranded option. Typically, about 92% of reads were mapped uniquely [except for D3(NMP-like) replicate 4, where uniquely mapped reads were at 86.8%]. Read counts per gene were found in the same STAR run. Data from Chu et al. (2016) were re-analysed in the same fashion; however, these were single-end non-stranded reads. For the following analysis, four biological replicates were used for D3(NMP-like) and two for D8 samples. Differential expression was performed with edgeR 3.16.5 for each pair of conditions independently. A Benjamini-Hochberg multiple-test correction was applied to test *P*-values. Human NMP-like genes (Fig. 3A) were determined by selecting genes using the following criteria: at least 10 read counts in D3(NMP-like), significantly enriched (*P*-value <0.01) in D3(NMP-like) compared with both hESC and hD8 samples, with a fold-change greater than 2. Time-dependent properties of genes were studied using intensity profiles hESC-D3(NMP-like)-D8. Each point in the profile is a DESeq-normalized mean gene count across replicates. To make profiles comparable, they were normalized to their mean across conditions, so the mean of each normalized profile is 1.

Comparisons between this human NMP-like signature and mouse NMP genes identified elsewhere was performed based on gene names. The gene list in Fig. 3C was obtained by comparing human NMP-like gene list (this study, full list in Table S1) and bulk-RNA-seq of mESC-derived NMPs (table S1 from Gouti et al., 2014). Gene lists in Fig. 3D were obtained by comparing the human NMP-like gene list (this study, full list in Table S1) and extended single-cell RNA-seq data obtained for NMP from E8.5 and E9.5 embryos (Gouti et al., 2017).

### Calcium imaging

To visualize Ca^2+^^ ^levels, D3(NMP-like) cells or D8 NPs (200K cells/cm^2^) were differentiated as described in Fig. 1B and incubated in a mixture of Fluo3AM (Invitrogen; stock 1 mM in DMSO, delivered to cells 1 μM) at 37°C for 30 min, rinsed with neurobasal medium (Gibco) supplemented as appropriate for D3 or D8 and left to recover for 1 h. Fluo3AM was then excited at 488 nm and the fluorescence generated was imaged by Deltavision Core microscope system in a WeatherStation environmental chamber maintained at 37°C. The D3(NMP-like) and D8 NP medium was buffered with a 5% CO_2_/95% air mix and maintained in a humid chamber. Images were acquired using an Olympus 20×1.30 NA objective using a Xenon light source and a CoolSnap HQ2 cooled CCD camera (Photometrics). Images were deconvolved and maximum intensity projections of *z*-stacks were made using SoftWorx imaging software (Applied Precision). To provide a positive control for response to calcium influx, D3(NMP-like) and D8 NP cells were incubated with A23187 (Sigma C7522) 10 μg/ml in 0.1% DMSO in neurobasal medium) at 37°C for 20 min, rinsed, incubated in with Fluo3AM for 30 min and then rinsed in neurobasal medium. The fluorescence generated was imaged as above. The raw data were then quantified using ImageJ plugin Heatmap Histogram. Data and statistical analyses are presented in Fig. S7 and its legend.

## Supplementary Material

Supplementary information
