# Supplementary material for: Neural differentiation, selection and transcriptomic profiling of human neuromesodermal progenitor-like cells in vitro
Source: Development. 2018 Jul 12;145(16):dev166215. doi: 10.1242/dev.166215 (PMC6124542; doi:10.1242/dev.166215)
Supplement: Supplementary information [file develop-145-166215-s1.pdf]

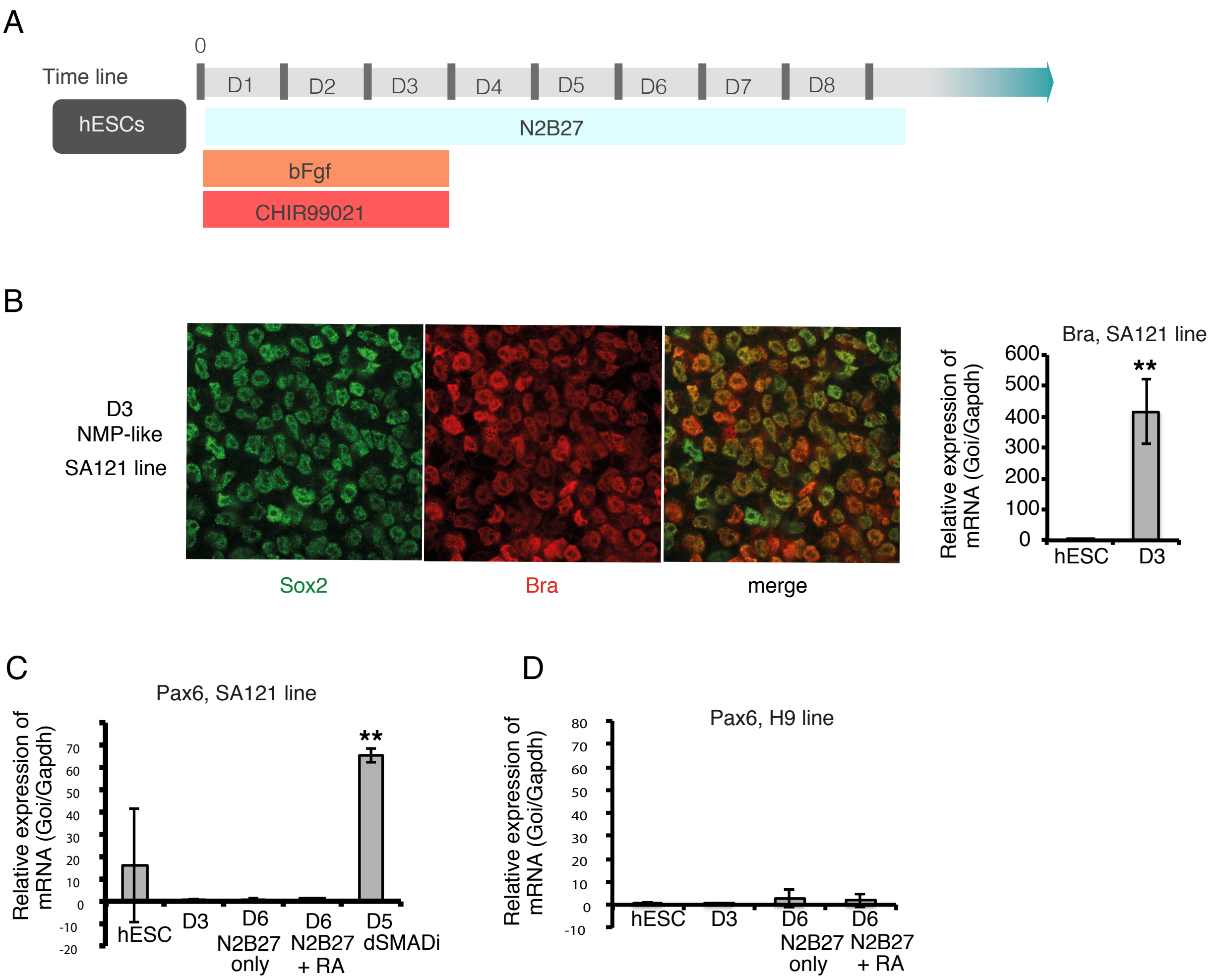

Figure S1Assessment of neural differentiation of hNMP-like cells in basic differentiation conditions

A. Basic protocol for in vitro generation and neural differentiation of hNMP-like cells as in (Gouti et al., 2014) and B. co-expression of Bra/Sox2 proteins on day 3 of protocol shown in A, detected by immunocytochemistry (3 independent experiments) and RTqPCR assessing relative expression of Bra during generation of NMP-like cells (SA121 line). C. RTqPCR for Pax6 in SA121 cell line cultured as indicated (SA121-line exhibits low-level Pax6 in hESC, while H9-line does not and so H9 was used for all subsequent experiments). D. RTqPCR for Pax6 in H9 cells cultured as indicated. This basic protocol did not elicit Pax6 expressing cells in either SA121 or H9 lines. This contrasted with the positive control for Pax6 transcription provided by a protocol for inducing anterior neural progenitors, exposure to Noggin 50 ng/ml and the TGF receptor type 1 inhibitor SB431542 10 M following removal of self-renewal conditions (dual SMAD inhibition, (Chambers et al. 2009), shown in C. RTqPCR graphs represent expression normalized to *Gapdh* and relative to hESC levels and all constitute 3 independent experiments, error bars are SEMs. Significant differences are represented here and for all subsequent RTqPCR data: ****p value <0.0001, ***p value<0.001, **p value<0.01, *pvalue<0.05 (ANOVA test, except for figure S1B:T-test).

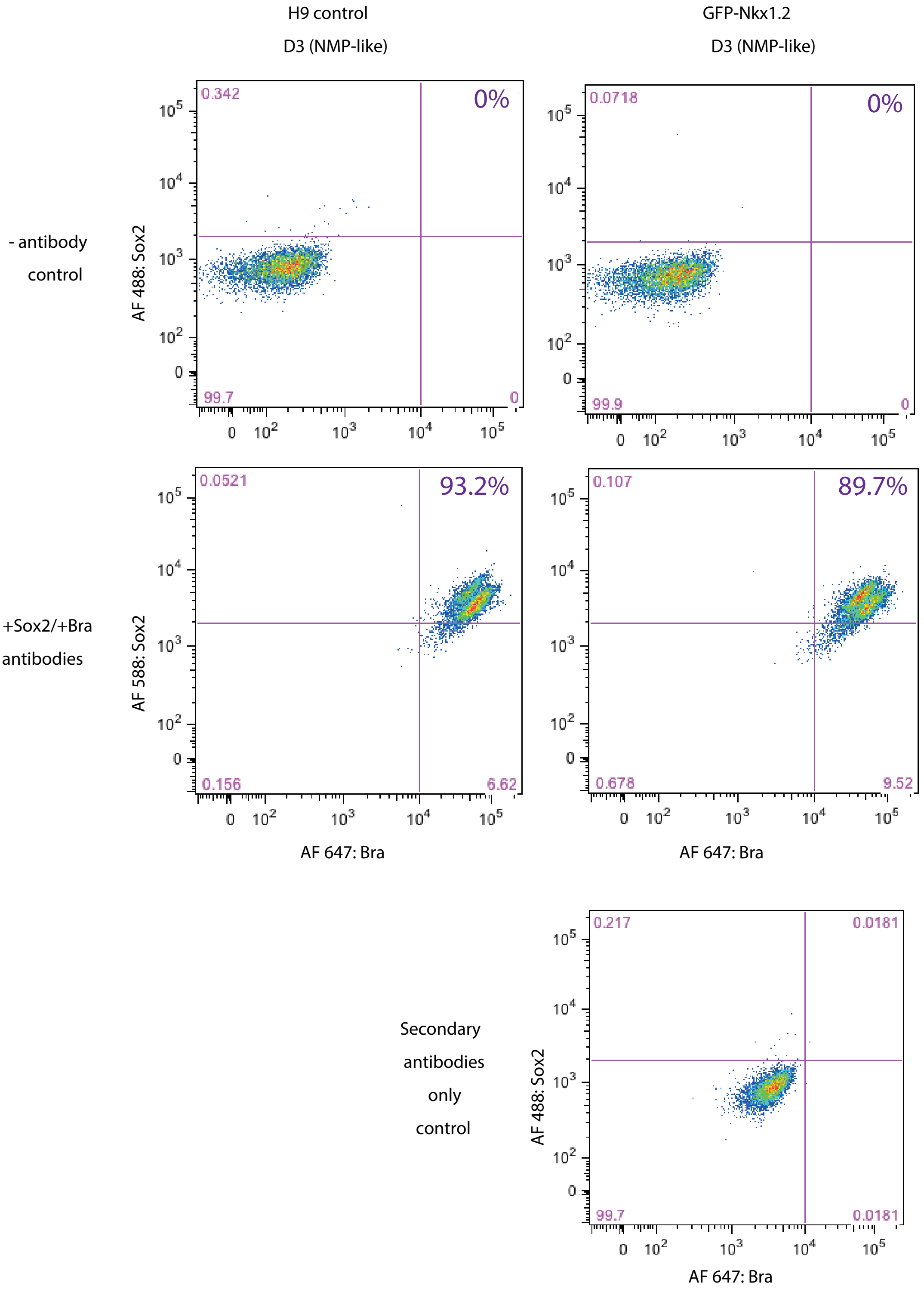

Figure S2 Co-expression of Sox2 and Bra proteins in hD3(NMP-like) cells

Expression of Sox2 and Bra was analyzed by flow cytometry in D3 cells derived from H9 cell line (left panels) and H9-GFP-Nkx1.2 cell line (right panels). Upper panels: no antibodies control, middle panels: staining with anti-Sox2 and anti-Bra antibodies, bottom graph: secondary antibodies alone (control). The quadrant for quantification of co-expression levels was defined based on fluorescence observed without primary antibody application (bottom graph). The percentage of co-expression for each panel is indicated at the upper right corner (purple). Representative experiment of 2 independent experiments.

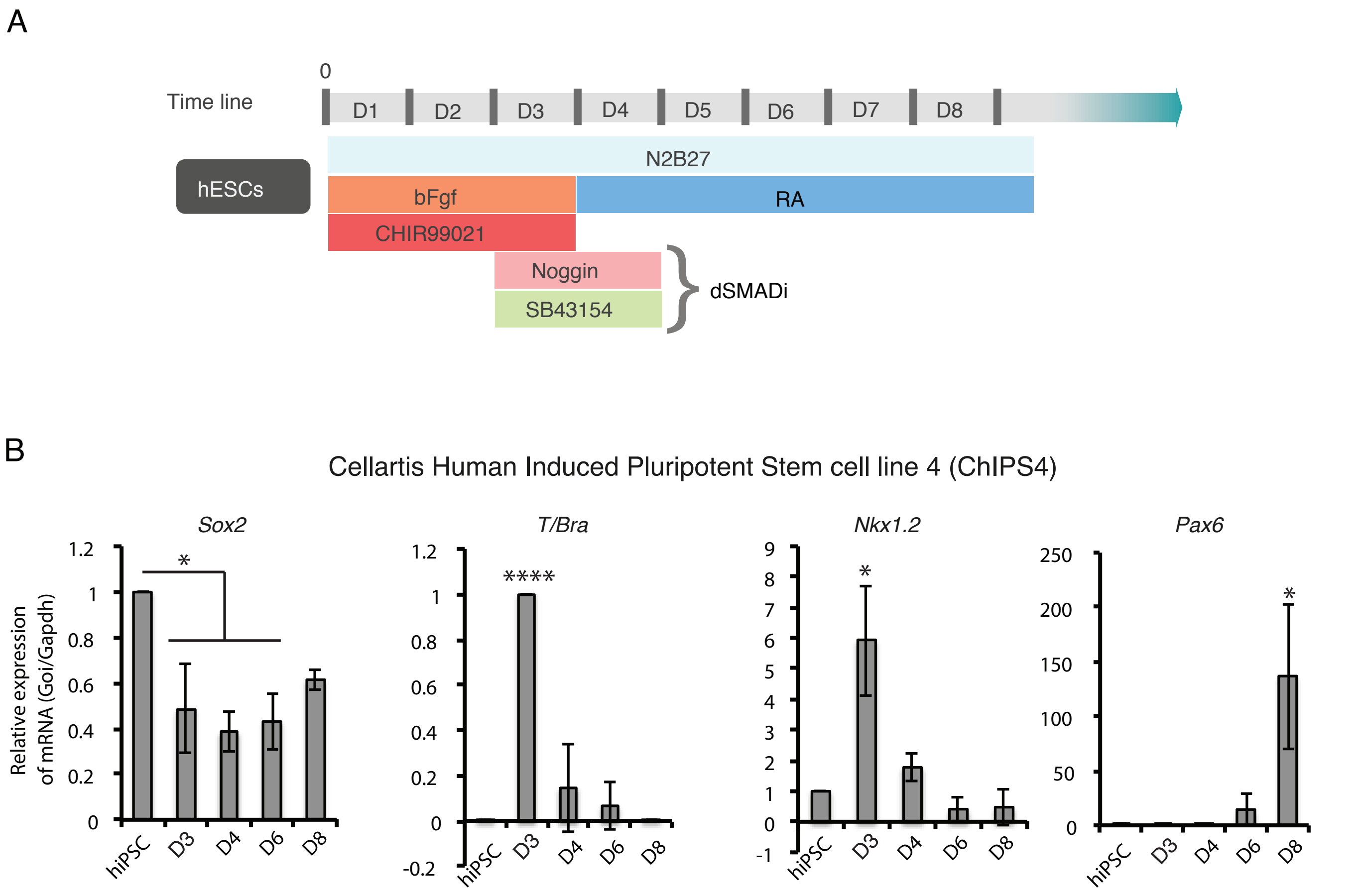

Figure S3 Robust differentiation of spinal cord progenitors from NMP-like cells in an iPS cell line

A. Schematic representation of the differentiation protocol used on the ChiPS4 cell line, including a dSMADi step from end of day 2 to end of day 4. B. Expression profile of selected genes over time in chiPS4 cells submitted to the differentiation protocol presented in A. Graphs represent the expression of each individual gene normalized to *Gapdh* and relative to hiPSC levels. RTqPCR data represents average of 3 independent experiments, error bars SEM.

Development • Supplementary information

AACCCTCCCTATAGGGGCGATTGGAGCTCCCGCGGTGCGGCCGCTCTAGAACTAGTG
GATCCCCCGGGCTGCAGCGACCAATGTGGAATTCGCCCTTAGTGGAAGCAAAAGACT
GAGAGTCCGGGATTTTCTCCCTCCGTTTCTGAGACAGCAGGATGTACTAAAAAAGCA
CTGACTGGTCCAGTAGAAGACCGAGGTCCAAACCCAGACTCTGTCACCAACTCACAG
TGACCCTGGGGAAATCTTTTCTTACCTTTGAACCTCGATTTCCTCATCTTTAAAACG
GGGACAGTGGTCTGTGCCACGTGACGCCCATCTCACAGGGATGCTCAAATAATCAAA
AGAGATCGTGCAAGCCTCAGGGCTTTGTGAACGCTAAACTGTGAGAACGTGAGGGAT
TTTACCTCCGAGGTAACCGGGTCTGAAGCTATTACAGTAATTCACTGGCGGGGAAGG
AGATGCGCTGAGCATTGCCTGGGAGTAAGCAGTCCTGGCCTCAGTTGCATCCCCAAG
TCTAAGGCGGGTGCACCGGAGAAAGGGAACAAACTCAAGTCACAGAGGTGTGTGTGT
CGGGGTGAGGGATCCCCGGGATGGAAGCCTCCCTCGCGCCCTCGGAGAGTCCCAGAG
GGTTGGGCGGAGGCGCGCGGAGACGACAACACTGTCCCCGCGGTCGCGCGCACCGGG
CGCGCGGAGGCTTCCCCGAGCCCAGGCAAGCGGCCGCGGCACAGCGCCTGATAGTCC
CGAGGCTGGCCCGGGCTGCGCCGGTGCCAATCGGCGCGCAGCCCCCCGCGGCGCTCT
CCCCGCCCCGCCTCCCCGCCCCCCTCCCCAGCTTCACTTGGCAGCGCGGACCCGGCT
CCTGGCTGGAAAGCTACCGCCAAGCCACAGCCGAAGGCAAGCCCGAGCGGCGCCATC
CCCAACCCCGCGCCGCCGACCGCCGGCCCGTGGGCGACGGGCATGGTGAGCAAGGGC
GAGGAGCTGTTCACCGGGGTGGTGCCCATCCTGGTCGAGCTGGACGGCGACGTAAAC
GGCCACAAGTTCAGCGTGTCCGGCGAGGGCGAGGGCGATGCCACCTACGGCAAGCTG
ACCCTGAAGTTCATCTGCACCACCGGCAAGCTGCCCGTGCCCTGGCCCACCCTCGTG
ACCACCCTGACCTACGGCGTGCAGTGCTTCAGCCGCTACCCCGACCACATGAAGCAG
CACGACTTCTTCAAGTCCGCCATGCCCGAAGGCTACGTCCAGGAGCGCACCATCTTC
TTCAAGGACGACGGCAACTACAAGACCCGCGCCGAGGTGAAGTTCGAGGGCGACACC
CTGGTGAACCGCATCGAGCTGAAGGGCATCGACTTCAAGGAGGACGGCAACATCCTG
GGGCACAAGCTGGAGTACAACTACAACAGCCACAACGTCTATATCATGGCCGACAAG
CAGAAGAACGGCATCAAGGTGAACTTCAAGATCCGCCACAACATCGAGGACGGCAGC
GTGCAGCTCGCCGACCACTACCAGCAGAACACCCCCATCGGCGACGGCCCCGTGCTG
CTGCCCGACAACCACTACCTGAGCACCCAGTCCGCCCTGAGCAAAGACCCCAACGAG
AAGCGCGATCACATGGTCCTGCTGGAGTTCGTGACCGCCGCCGGGATCACTCTCGGC
ATGGACGAGCTGTACAAGTCCGGACTCGGATCC**GAGGGCAGAGGAAGTCTTCTAACA**
TGCGGTGACGTGGAGGAGAATCCCGGCCCACTGGCATGGCAaGAtGGtGGtGCCAAG
GCGGCTCCCTCCCACCACAAGATTTCTTTCTCTGTCCTGGACATCCTGGACCCACAG
AAATTCACCCGCGCAGCGCTCCCTGCCGTGCGCCCGGCTCCCCGGGAAGCCAGGAAA
AGTTTGGCGGAGGTCGAAGCGGGGAAAGATGCCAGCTCCAGGGACCCTGTCCGACAG
CTGGAGACCCCTGGTAAGATGCAAGGCGGCCCCGGCCCCAGGAGGCCTCAGCCCCAA
CAATGCGGAGTGTATGGGGGAACAGCCGGGCCCGGTGAGTGGCCCTTAACAGCGTCT
TCCTCAGAGAGAAGGCGCACGGGACCGGGTGCGAAGTGTAGCCCCCGCCTCGGACTT
GGATAGAGGCAGAGAGGAGGCTCCCCGCATTACAGGGCAGGGATTTGCCGCATCCCT
GCTCACCCGCCAAGCTCACCCGCACCACAGTTCTGATGCTCGCGGTGGAAACTTACC
TGGCGCCTGTCTTGCCAGGCTTACTCATTTATCGGGCATTTAATGCGCTTGCCACGT
GCTAGGACCTGGGCTAAGGGCTGGGATAAAGGTGATGAAAACTTCGGAACCTGAGAG
ATGGACAGCATCATTAACATCACCTCCATTTTATGGATGGGGGAGCTGACGCTAAGG
CTTGCGCCGGGGGTCTTCGTGAGTAGCGAGGTCAGGTGCCACCCGGAGACGCCTGCG
GGGCTGGGCTGCCCCAGGCGCTCAAGCTCCCCAGACTCAAC**TGCCCGCTACCTCGAG**
GGCGCCACGCCGGCGAGATCTTGATCACCTAGGGGGCCCGACGTCGCTGGTTACACC
TTAAGCGGGAA

Development • Supplementary information

Legend:

Plamid sequence
Primers used for cloning
gRNA (antisense and sense)
GFP sequence
T2A sequence
Nkx1.2 Exon1
repeats

Figure S4
Sequencing of the GFP-T2A insertion site in the correctly targeted clone used in this study. Grey: plasmid sequence, highlighted yellow: Primers used for cloning, highlighted blue: gRNA (antisense and sense), highlighted green: GFP sequence, orange: T2A sequence, bold black: Nkx1.2 Exon1, peach: repetitive sequences.

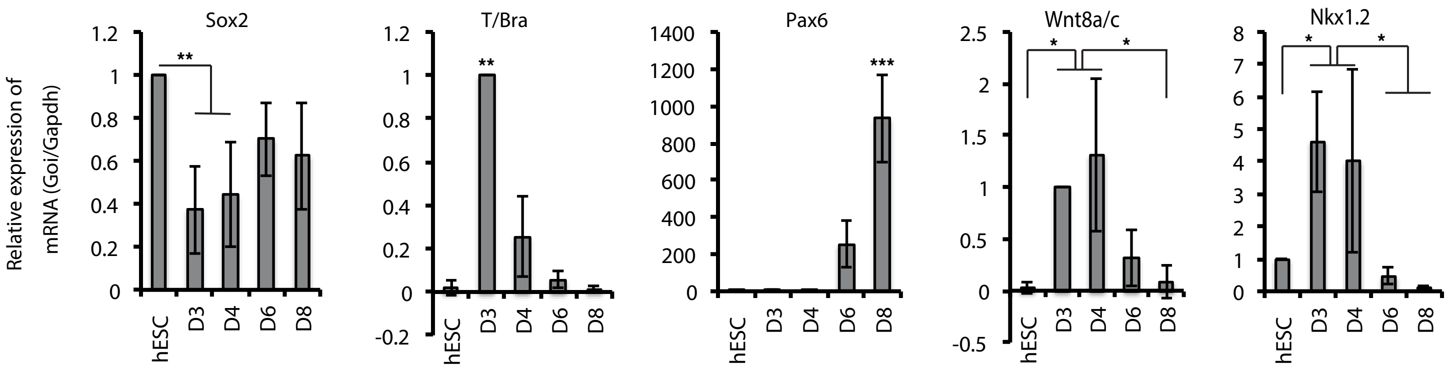

Figure S5 Diffentiation evaluation of the GFP-Nkx1.2 clone used in this study

Expression of selected marker genes was analyzed by RTqPCR during differentiation of the GFP-Nkx1.2 line following the protocol presented Figure 1B. Graphs represent the expression of each individual gene normalized to *Gapdh* and relative to hESC levels. Average of 3 independent RTqPCR experiments, error bars are SEM.

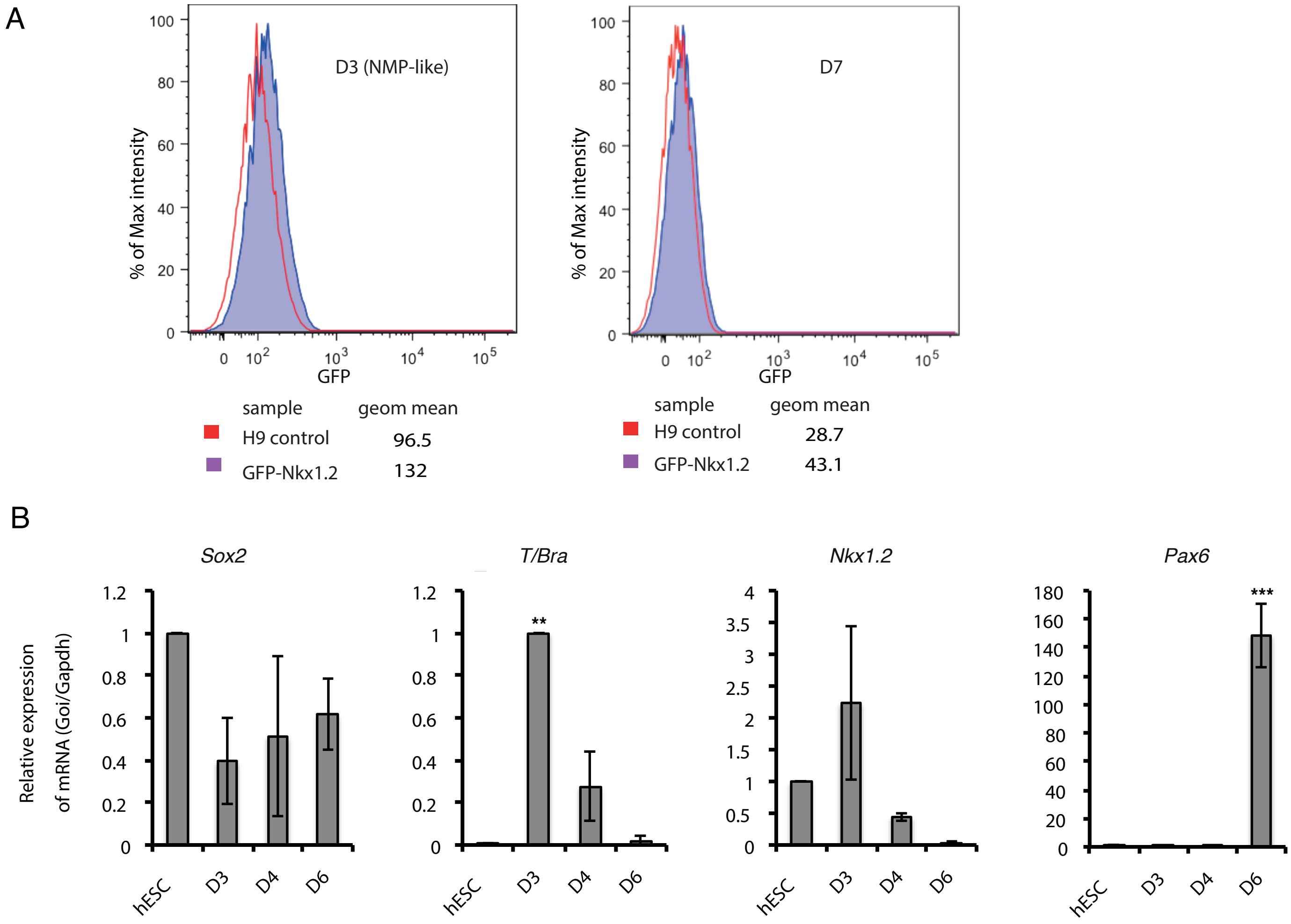

Figure S6 GFP expression and differentiation profile of a second correctly targeted GFP-Nkx1.2 clone

A. Flow cytometry analysis of GFP expression at D3 (NMP-like) and D7 of the differentiation protocol. % of maximum intensity for GFP channel is plotted, geometric mean for each peak is indicated. B. Expression of selected marker genes analyzed by RTqPCR during differentiation of the second GFP-Nkx1.2 clone following the protocol presented Figure 1B. Graphs represent the expression of each individual gene normalized to *Gapdh* and relative to hESC levels. Average of 3 independent RTqPCR experiments, error bars are SEM.

Development • Supplementary information

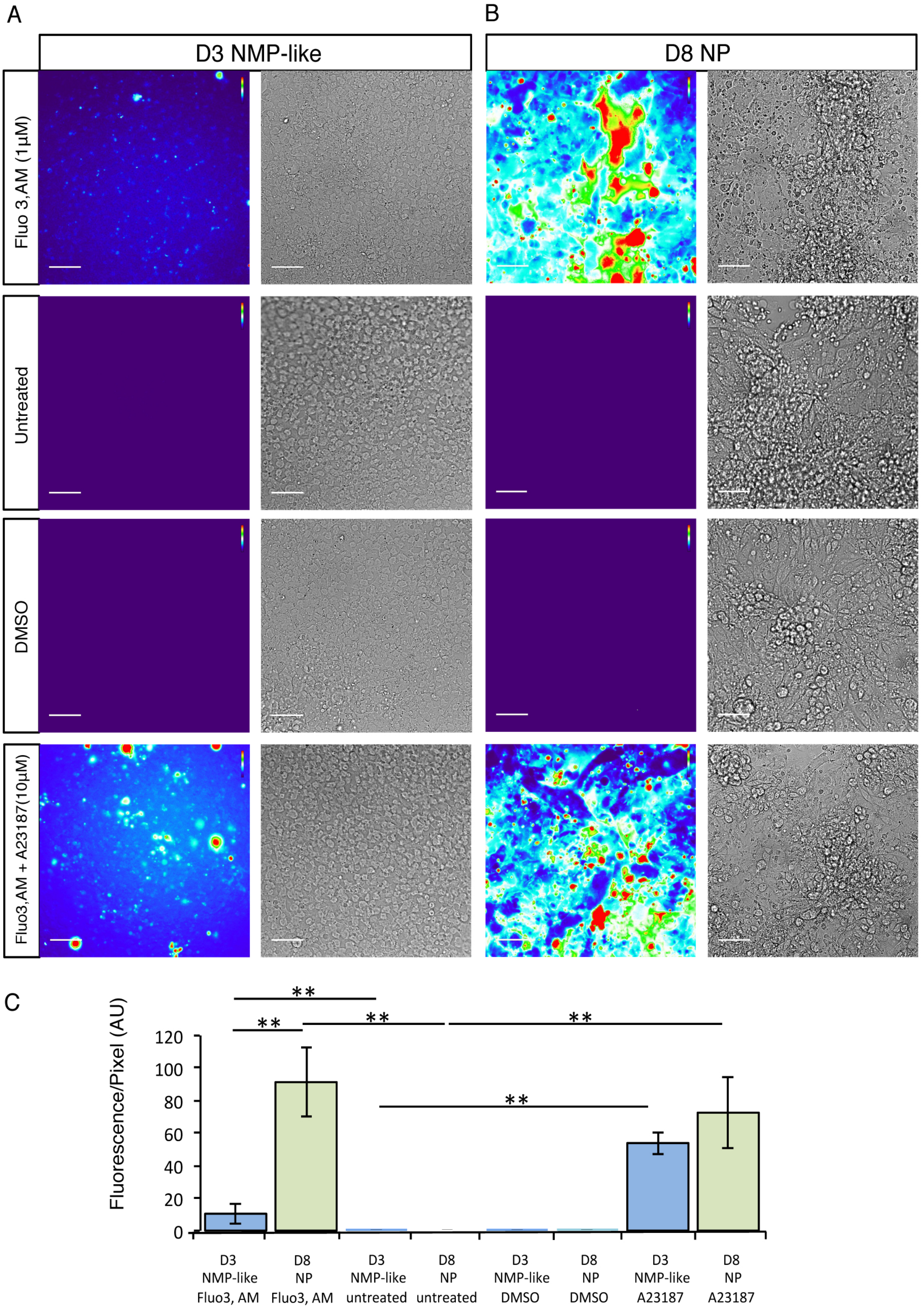

Figure S7 Calcium signalling increases as NMP-like cells differentiate into neural progenitors

Calcium signalling was assessed using Fluo-3, AM in D3 NMP-like cells and in D8 neural progenitors (NP). A. D3 NMP-like cells exposed to Ca^{2+} indicator, Fluo-3, AM (in DMSO/medium), medium alone, medium with vehicle DMSO alone, or the calcium ionophore A23187 in the presence of Fluo-3, AM; B. D8 NPs treated with Fluo-3, AM (in DMSO), medium alone, medium with vehicle DMSO alone, or the calcium ionophore A23187 in the presence of Fluo-3, AM. Green emission of Fluo-3, AM excited at 488 nm has been pseudo-coloured and presented as a heat-map using the HeatMap Histogram plugin for Image J (red=high and blue=low fluorescence); C): Quantification and comparison of calcium fluorescence in D3 NMP-like cells and D8 NPs exposed to Fluo-3, AM, medium alone, medium with DMSO or the calcium ionophore A23187 in presence of Fluo-3, AM. Quantification was made using the total fluorescence intensity from 3 images for each condition from 4 independent experiments. Data were analysed using the non-parametric Mann-Whitney test with Graphpad Prism V6. Error bars are ± standard deviations. p-value $^{**}p<0.01$. Scale bar = 50 µm. These data show that calcium signalling is higher in D8 neural progenitors in comparison with D3 NMP-like cells from which they are derived.

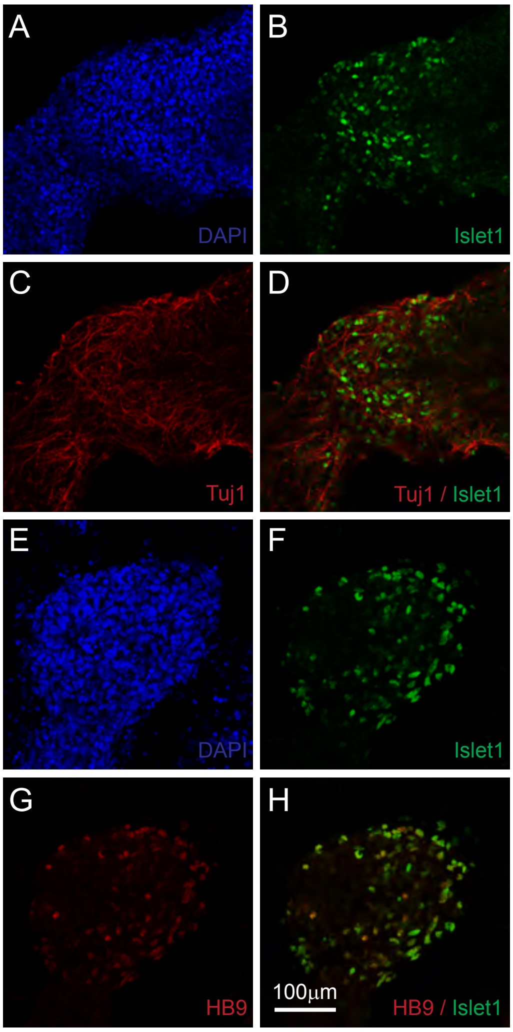

Figure S8 Motorneuron differentiation from hESC derived NMP-like cells

H9 ES cells were differentiated into neuromesodermal progenitor-like cells as in Fig1B and then differentiated towards motor neurons as described in the Methods. Nuclei were stained with DAPI (A and E), and labelled with antibodies against Islet1 (B,D,F and H), beta-III-tubulin (C and D) and HB9 (G and H). Motorneurons were identified as cells co-expressing HB9 and Islet1 (Amoroso et al. 2013). Images are representative of cells cultured using this protocol in 3 independent experiments starting from the hES cell state. Scale bar = 100 µm

Supplementary Materials and methods

Quality control and passage numbers for pluripotent cells used in this study

H9 (WA09) hES cells were purchased from Wicell and were supplied at passage 24. The cells were thawed transferred to DEF-CS and cell banks prepared at passage 29. For routine production the cells were used between passage 29 and 39.

SA121 hES cells were purchased from Cellartis AB and were supplied at passage 9. The cells were thawed and cell banks prepared at passage 13. For routine production the cells used between passage 13 and 23.

ChiPS4 hiPS cells were purchased from Cellartis AB and were supplied at passage 9. The cells were thawed and cell banks prepared at passage 13. For routine production the cells used between passage 13 and 23.

For making the Nkx1.2 GFP knock in line H9 (WA09) cells were transfected at passage 33 and monoclonal cell lines banked at passage 40. For routine production the cells used between passage 40 and 50

For quality control purposes, representative lots of each cell bank were thawed and tested for post-thaw viability, and to ensure sterility and absence mycoplasma contamination. After 2 passages the cell lines were tested for the expression of pluripotency markers (Oct4, Sox2, Nanog, SSEA-3, SSEA-4, TRA-1-60 and TRA-1-81) and differentiation markers (SSEA-1, HNF-3 beta, beta-III-tubulin and smooth muscle alpha-actinin) by immunofluorescence, and the ability to form all three germ layers when embryoid bodies are allowed to spontaneously differentiate in culture (immunofluorescence for HNF-3 beta, beta-III-tubulin and smooth muscle alpha-actinin).

Table S1.

List of genes specifically enriched in human NMP-like cells.

Human NMP-like genes (Fig. 3A) were determined by selection of RNAseq data using criteria indicated in the methods section (at least 10 read counts in D3(NMP-like), significantly enriched (p value < 0.01) in D3(NMP-like) compared to both hESC and hD8 samples, with a foldchange >2). Using these criteria, 1348 genes were identified as highly expressed in human NMP-like cells (D3). Full list including information on the 1348 hNMP-like genes are included in sheet 1. Sheet 2 contains the selected dataset used to make figure 3A. Both tables present for each gene: gene names and description, mean read counts from independent experiments in hESC, D3(NMP-like) and D8, fold change between (D3)NMP-like and hESC conditions (FC NMP-like/hESC), fold change between (D3)NMP-like and D8 conditions (FC NMP-like/D8), and p-values associated (p_hECS.NMPlike, p_NMPlike.D8, p_hESC.D8).

Click here to Download Table S1

Table S2. Primers for qPCR

Click here to Download Table S2
